# Use of Extraocular Muscle Flaps in the Correction of Orbital Implant Exposure

**DOI:** 10.1371/journal.pone.0072223

**Published:** 2013-09-25

**Authors:** Hsueh-Yen Chu, Yi-Lin Liao, Yueh-Ju Tsai, Yen-Chang Chu, Shu-Ya Wu, Lih Ma

**Affiliations:** 1 Department of Ophthalmology, Chang Gung Memorial Hospital, Linkou, Taiwan; 2 College of Medicine, Chang Gung University, Taoyuan, Taiwan; Duke University, United States of America

## Abstract

**Purposes:**

The study is to describe a new surgical technique for correcting large orbital implant exposure with extraocular muscle flaps and to propose a treatment algorithm for orbital implant exposure.

**Methods:**

In a retrospective study, seven patients with orbital implant exposure were treated with extraocular muscle flaps. All data were collected from patients in Chang Gung Memorial Hospital, Taiwan during 2007–2012. All surgeries were performed by one surgeon (Y.J.T). Patient demographics, the original etiology, details of surgical procedures, implant types, and follow-up interval were recorded. Small exposure, defined as exposure area smaller than 3 mm in diameter, was treated conservatively first with topical lubricant and prophylactic antibiotics. Larger defects were managed surgically.

**Results:**

Seven patients consisting of two males and five females were successfully treated for orbital implant exposure with extraocular muscle flaps. The average age was 36.4 (range, 3–55) years old. Five patients were referred from other hospitals. One eye was enucleated for retinoblastoma. The other six eyes were eviscerated, including one for endophthalmitis and five for trauma. Mean follow-up time of all seven patients was 19.5 (range, 2–60) months. No patient developed recurrence of exposure during follow-up. All patients were fitted with an acceptable prosthesis and had satisfactory cosmetic and functional results.

**Conclusions:**

The most common complication of orbital implant is exposure, caused by breakdown of the covering layers, leading to extrusion. Several methods were reported to manage the exposed implants. We report our experience of treating implant exposure with extraocular muscle flaps to establish a well-vascularized environment that supplies both the wrapping material and the overlying ocular surface tissue. We believe it can work as a good strategy to manage or to prevent orbital implant exposure.

## Introduction

Orbital implant was developed for orbital volume replacement after the evisceration or enucleation surgery. The first orbital implant was used in 1885. [1] In 1985, the porous hydroxyapatite orbital implant was introduced and became increasingly popular in the anophthalmic socket management. [2] Fibrovascular ingrowth through the pores into the biocompatible implant can increase motility and prevent its migration or extrusion. Wrapping the implant decreases the irritating effects of uneven surface and also reduces implant exposure, migration or extrusion. [3].

The most common complication of orbital implant is exposure, caused by breakdown of the covering layers, leading to extrusion. [4] Several methods were reported to manage the exposed implants, including scleral patch grafts, [5] mucous membrane grafts, [6] temporalis fascia grafts, [4] conjunctival pedicle grafts [7] and dermis fat grafts. [8]–[10] In an effort to manage exposure, extra-ocular muscles were explored and detached from the wrapped implant and sutured onto the exposed area after filling up the defect with banked sclera or fascia lata. In this way, the extraocular muscle acted as the vascular bed, supplying both the underlying wrapping tissue and the overlying ocular surface tissue, such as oral mucosal graft, tenon tissue and conjunctiva.

## Materials and Methods

### Ethics Statement

Chang Gung Medical Foundation Institutional Review Board (IRB) approval was obtained with the designated IRB number: 102-0044B.

All data were collected from patients in Chang Gung Memorial Hospital, Taiwan during 2007–2012. The Chang Gung Medical Foundation IRB had waived the written consent given by the patients or by the next of kin, caretakers, or guardians on the behalf of the minors/children participants for their information to be stored in the hospital database and used for research. For it was a retrospective chart review study without human tissue sample being obtained and the privacy of the patients was protected without identifiable photos of the patients being used.

Five of seven patients were referred from other hospitals. A retrospective medical record review was carried out. Patient demographics, the original etiology, details of surgical procedures, implant types, and follow-up interval were recorded. Small exposure, defined as exposure area smaller than 3 mm in diameter was treated conservatively first with topical lubricant and prophylactic antibiotics. Larger defects were managed surgically.

### Surgical Techniques

All surgeries were performed by one surgeon (Y.J.T) under general anesthesia. Retrobulbar injection with 1∶1 mixture of 1% lidocaine with 1∶100000 epinephrine and 0.75% bupivicaine. After sterile prepping and draping, 360 degrees periotomy was performed. Care was taken to dissect between tenon and orbital implant.

#### In patients with porous implants (Figure 1)

The previous implants were inspected for integrity and infection. Carefully dissect the conjunctiva from the wrapped implant. Infected or rugged orbital implants were exchanged with new bioceramic ones. The extraocular rectus muscles adjacent to the exposure area were isolated, particularly the horizontal recti. Isolated rectus muscles were sutured over the exposure area with 6-0 vicryl after the defect was covered by the sclera or wrapping material.

#### In patients with non-porous implants (Figure 2)

The previous wrapped implants were enucleated after the six extraocular muscles were detached. The non-porous implants were exchanged with new bioceramic ones. Envelop the implant with sclera, fascia lata or original wrapping material. Insert the new implant back to the socket with a small naked area of implant surface turned toward the orbital apex, acting as a vascular window for fibrovascular ingrowth into the new implant. Isolated rectus muscles were first sutured with 6-0 vicryl onto the wrapping material, followed by oblique muscles for coverage augmentation.

**Figure 1:**
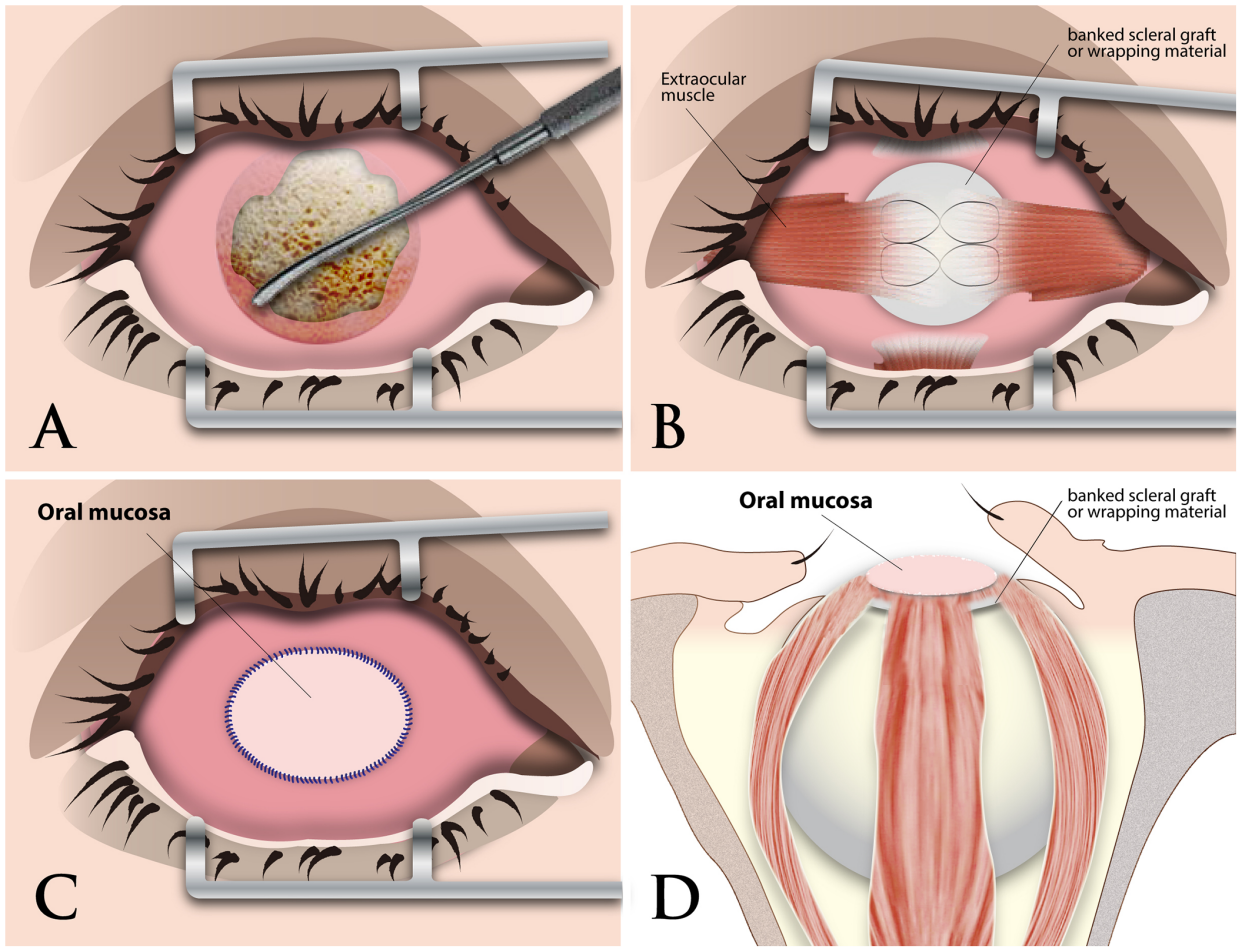
Surgical procedures of orbital implant exposure management on porous implants. (A) Carefully dissect along the subtenon and orbital implant and check the extension of exposure area; (B) Suture the four isolated extraocular rectus muscles onto the wrapped implant; (C) Harvest oral mucosa after sizing the conjunctival defect and suture the harvest oral mucosa to the surrounding conjunctiva of the ocular surface; (D) The sagittal view of the orbit illustrates the relative positions of the implant, the wrapping material, extraocular muscles, and the oral mucosa.

**Figure 2:**
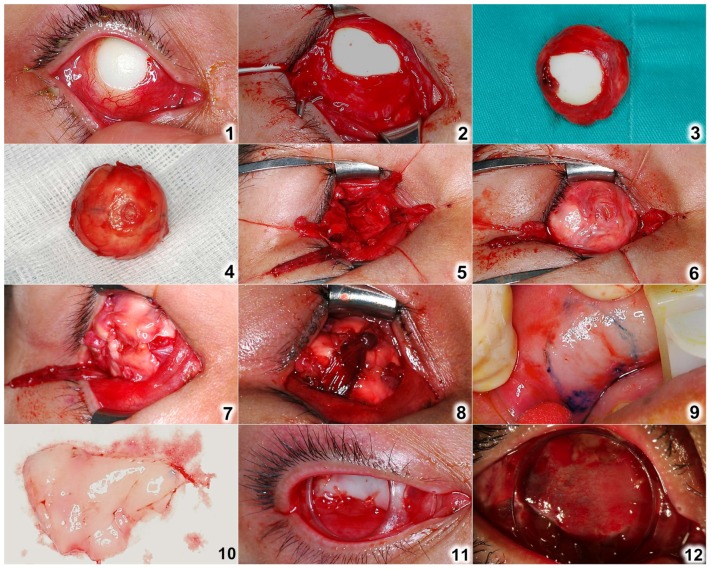
Surgical procedures of orbital implant exposure management on non-porous implants. (1) Document the appearance of the exposed implant; (2) Carefully dissect between the tenon and the orbital implant; (3) Enucleate the wrapped implant (front); (4) Inspect for irregularity or infection and invert the implant; (5) Isolate six extraocular muscles; (6) Insert the wrapped implant with the exposure surface facing toward the orbital apex; (7) Suture the rectus muscles onto the wrapped implant; (8) Reinforce with oblique muscles to augment coverage; (9) Harvest oral mucosa after sizing the conjunctival defect; (10) Suture the harvest oral mucosa to the surrounding conjunctiva of the ocular surface; (11) Insert conformer to maintain fornices for future prosthesis fitting; (12) Inspect the wound regularly (post-operative 10 days).

After sizing the conjunctival shortage on the ocular surface with upper and lower fornices depth being maintained, oral mucosa was harvested and sutured to conjunctiva surrounding the defect with 6-0 vicryl. Ring conformer was inserted at the end of the surgery. Topical antibiotics ointment was dressed. Pressure patch was placed for two days. Procedures were illustrated in Figure 1 for porous implants and Figure 2 for non-porous implants, respectively.

## Results

Seven patients consisting of two males and five females were successfully treated for orbital implant exposure with extraocular muscle flaps. The average age was 36.4 (range, 3–55) years old. Five patients were referred from other hospitals. One of seven eyes was enucleated for retinoblastoma. Mesh-wrapped bioceramic implant was inserted. The other six eyes were eviscerated, including one for endophthalmitis and five for trauma. Autogenous scleral grafts were used in four of six patients as wrapping material, while glycerol cornea and autogenous fascia lata were patched on the other two patients respectively. Two of seven impants were exchanged by new ones. One of them was due to implant malplacement with irregular surface and proptotic appearance (patient 4); the other one was because the primary implant had been avascular non-porous silicon ball (patient 7). Three patients (patient 1,2,5) had peg fitted into the orbital implant.

### Clinical Report

#### Patient 1

A 55-year-old female received hydroxyapatite implant insertion with peg system after evisceration. Peg loosening was noted 4 years later with small exposure area. Histoacryl blue glue (TissueSeal, Ann harbor, Michigan) was used to stabilize the peg, but conjunctiva recession occurred, resulting in enlarged exposure measuring 13 mm in diameter. We covered the exposure area with banked sclera graft first, but the sclera melted 1 month later. We attributed the failure to poor vascular supply and covered the exposure with orbicularis bi-pedicle muscle flap but it failed to recover either. The reason was presumed to be thin flap without adequate vascular supply. Hence we used extraocular muscle flaps to cover the exposure area and it was successfully treated.

#### Patient 2

A 53-year-old female was referred to our clinic due to persistent yellowish brown discharge for nearly a year. Her implant had been pegged twice with the plastic one and the titanium one sequentially. The exposed hydroxyapatite was repaired with the advancement of extraocular muscles. We treated micro-infection without implant extraction by repeated antibiotics injection into the retro-implant space and by embedding antibiotics-mixed bone graft substitute into the peg hole. The conjunctiva eventually grew into the pegging hole.

#### Patient 3

The left eye of a 3-year-old girl was enucleated due to retinoblastoma. She was referred for exposure found during follow-up. Muscle slippage was noted in the surgical exploration and was considered the main cause of exposure. We successfully managed the exposure by using only one intra-operatively identifiable superior rectus muscle for the re-establishment of the vascular bed.

#### Patient 4

A 35-year-old male complained of persistent discharge and eye size asymmetry with the artificial eye bigger than the normal one a year after evisceration. Chronic inflammation with implant exposure and a rather proptotic appearance was found in the injured eye. During the revision surgery, we noticed the malplacement of orbital implant and decided to replace the implant with a smaller one by 2 French units (Fr.). Extraocular muscles were advanced to cover the exposure. The patient was satisfied with the cosmetic appearance after the surgery and no exposure occurred to date.

Mean follow-up time of all seven patients was 19.5 (range, 2–60) months. No patient developed recurrence of exposure during follow-up. All patients were fitted with an acceptable prosthesis and had satisfactory cosmetic and functional results. Detailed demographic data were summarized in Table 1.

**Table 1:** Demographics of patients.

Patient	Age/sex	Primary operation	Pre-operativediagnosis	Post-op Exposuretime	Correctiontime*	Original Implant/size (Frenchunit, Fr. ¶)	Wrapping material	Implant exchange	Follow-up time
1	55/F	evisceration	trauma	48 months	2010	Hydroxyapatite with Peg/18 Fr.	sclera	No	28 months
2	53/F	evisceration	trauma	180 months	2011	Hydroxyapatite with peg/unknown	glycerol cornea	No	13 months
3	3/F	enucleation	retinoblastoma	26 months	2011	Mesh-wrapped bioceramic/18 Fr.	none	No	13 months
4	35/M	evisceration	trauma	5 months	2012	Hydroxyapatite/20 Fr.	sclera	To 18 Fr. bioceramic	7 months
5	23/F	evisceration	trauma	36 months†	2011	Hydroxyapatite with peg/20 Fr.	Autogenous fascia lata	No	14 months
6	48/F	evisceration	endophthalmitis	0.75 months	2007	Bioceramic/unknown	sclera	No	60 months
7	38/M	evisceration	trauma	180 months	2012	Silicon/18 Fr.	sclera	To 20 Fr. bioceramic	2 months

* Correction time means the time of surgery to correct implant exposure;

¶ 1 Fr. = 0.33 mm.

† The patient lost follow-up after the surgery. Implant exposure was noted at the time of return visit.

Naked bioceramic implants were used unless specified as mesh-wrapped biocermaic.

## Discussion

The risk factors of orbital implant exposure can be divided into early and late onset. Early conjunctival thinning or erosion is noted in the first few weeks postoperatively. It is related to previous surgery, trauma, radiotherapy or secondary implantation and is mainly due to inadequate wound closure. [5], [11] Late defect occurs months after the surgery and is due to pressure from the prosthesis to the conjunctival surface causing necrosis, spicules on the porous implant surface inducing chronic irritation and excessive inflammation associated with implants, anterior orbital misplacement, or lack of implant vascularization. [10], [12] The rate of implant exposure varies significantly by implant types, with porous polyethylene and aluminum oxide having the greatest proportion. [13] Hence, patients with orbital implants should be regularly followed on a long-term basis to detect the complication. In our study, patients with enormous exposure to the equator are those who loses follow-up. Peg system is also reported to play a role in the increased rate of late-onset complications, exposure and infection. [14] Three of our patients have peg fitted in the orbital implant. One of them presents with loosened peg and fragile coral. Micro-infection persists for several months and is controlled without implant extraction by repeated retro-implant antibiotics injection and antibiotics-mixed bone graft substitute embedded into the peg hole. As the treatment of infection takes time and efforts, we think it would be better to exchange the infected implant directly in the management of infected implant exposure.

Various materials, such as sclera, dura, amniotic membrane and mesh [2] were used to wrap porous implants to reduce surface friction and to facilitate implant insertion. It also acted as an additional layer of barrier to resist implant exposure and extrusion. Autogenous materials that have been used include fascia lata, [15] temporalis fascia, subconjunctival flap, retroauricular myoperiosteal graft, [16] and posterior auricular muscle complex grafts. [3] Dermis fat graft was also used in concurrent volume and conjunctival insufficiency. They were thought to be less antigenic immunologically and free from allogenic transmission of infectious diseases. [3].

The fibrovascular ingrowth into the orbital implant from the adjacent tissue is important in keeping the implant in proper position and thus prevents exposure or extrusion. The vascular supply to the anterior part of implants forms the vascular beds for the overlying tissue, such as tenon and conjunctiva to survive. [9] Holding this concept, we advanced the extraocular muscle pedicles onto the exposed porous orbital implant to enhance the vascular supply. Extraocular muscles provide abundant vascular loam for the overlying tenon and conjunctiva, along with the underlying added wrapping material, to thrive on. For the cases of exposed non-porous implant, both rectus and oblique muscles were used after implant exchange. We firstly sutured rectus muscles onto the wrapped implant, followed by oblique muscles to expand the coverage. Use of inferior oblique muscles has been reported in augmenting implant coverage in enucleation surgery. The muscle belly averages about 7 mm in width, providing a robust portion of tissue to cover the anterior aspect of the implant that is most susceptible to exposure. [17] We exchanged the non-porous silicone implant with porous bioceramic in one of our cases to increase as much as possible the blood supply to nourish the anterior portion of the implant as well as the ocular surface tissue, preventing conjunctival thinning, erosion and eventual implant exposure.

We used the oral mucosa graft with great elasticity in cases of severe exposure accompanied by insufficient conjunctiva to maintain fornical depth for future surface prosthesis wearing. The graft can survive well only with the help of well-vascularized environment provided by the fibrovascular ingrowth from the implant and by the extraocular muscle pedicles. Burring the anteriorly avascular part of the exposed implant is reported as a gold standard for reestablishing the vascularized environment to prevent re-exposure. [8] With this technique, we do not have to burr away the avascular part of the porous implant but are still able to rebuild the ocular surface, which enables larger orbital volume for cosmetic acceptance. Another advantage of using extra-ocular muscle flaps is its vicinity to the orbit, which is familiar to oculoplastic surgeons. Adding to that, no additional visible surgical scars occur with this technique.

Most of the patients in our study previously received evisceration rather than enucleation. This can be explained by the differences of surgical techniques. In primary evisceration, extraocular muscles are seldom pulled forward to cover the implant surface as opposed to enucleation, in which extraocular muscles are generally sutured to the anterior portion of the orbital implant. It further supports the presumed mechanism that good vascularization plays an important role in the wound recovery and in the prevention of orbital implant exposure. [10].

**Figure 3:**
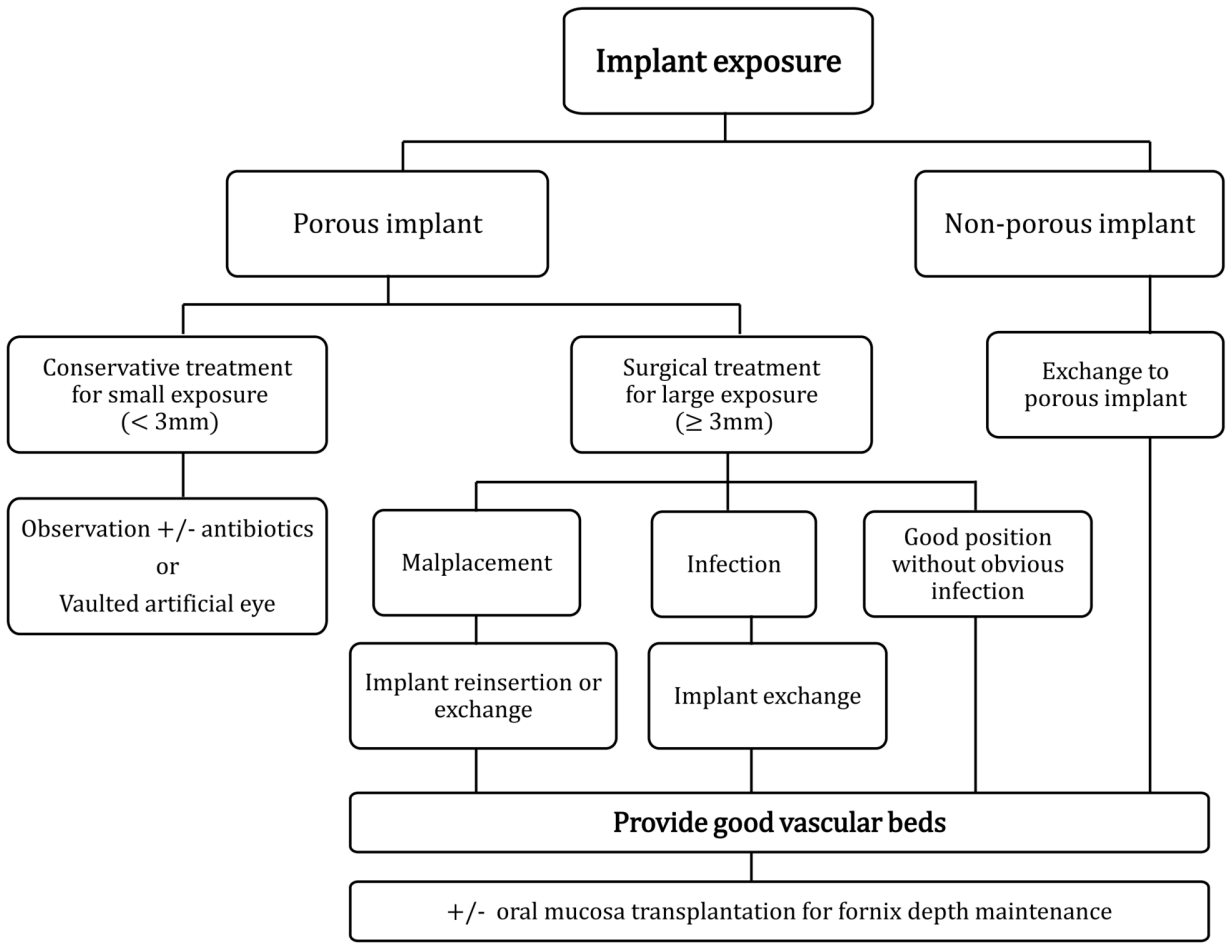
Algorithm for managing the implant exposure. Different approaches are applied on different implant types. For porous implants, exposure smaller than three millimeters is treated conservatively. Larger exposure is surgically managed according to the respective mechanism. For non-porous implants, exchange the implants to porous ones. Good vascularization is universally provided. Oral mucosa is transplanted when the remaining conjunctiva is incapable of supporting fornices adequately.

Based on this study, we propose a treatment algorithm for orbital implant exposure. (Figure 3). For non-porous implant exposure, exchanging the implant to porous one can enhance the stability due to fibrovascular ingrowth. For porous implant exposure, conservative treatment with topical lubricants and prophylactic antibiotics can be enough in exposure smaller than 3 mm. Causes of exposure should be sought and treated. Artificial eye can be vaulted to reduce contact irritation. In cases of larger exposure, several differentials including infection and malplacement should be corrected. If implant position is good without obvious infection, poor vascularization should be suspected. Techniques described in our study can be implemented to solve the problem. The limitation of the study is its small case numbers. However, all these patients received prior surgeries complicated with implant exposure, which rendered them difficult to handle. Because our technique successfully solved the problem, we believe it can be applied to patients requiring primary enucleation or evisceration surgeries to prevent subsequent implant exposure.

Orbital implant exposure is a potential complication in enucleation and evisceration surgery [13]. Several methods have been addressed on its management. We report our experience of treatment with extraocular muscle flaps to establish a well-vascularized environment that supplies both the wrapping material and the overlying ocular surface tissue, such as oral mucosa or conjunctiva. We believe it can work as a good strategy to manage or to prevent orbital implant exposure.
