# Correction: Use of Extraocular Muscle Flaps in the Correction of Orbital Implant Exposure

**DOI:** 10.1371/annotation/d77a80cc-f8bc-4140-b0b3-c9f05c861776

**Published:** 2013-11-15

**Authors:** Hsueh-Yen Chu, Yi-Lin Liao, Yueh-Ju Tsai, Yen-Chang Chu, Shu-Ya Wu, Lih Ma

The Publication Date given in the PDF version and expressed in the Citation and Footer in the PDF version of the article is incorrect. The correct publication date is September 25, 2013. 

The correct Citation is: Chu H-Y, Liao Y-L, Tsai Y-J, Chu Y-C, Wu S-Y, et al. (2013) Use of Extraocular Muscle Flaps in the Correction of Orbital Implant Exposure. PLoS ONE 8(9): e72223. doi:10.1371/journal.pone.0072223. The Footer in the bottom right corner of each page of the PDF should read: 2013 | Volume 8 | Issue 9 | e72223. 

